# Solidarity, justice, and recognition of the other

**DOI:** 10.1007/s11017-016-9387-3

**Published:** 2016-11-28

**Authors:** Ruud ter Meulen

**Affiliations:** Centre for Ethics in Medicine, School for Social and Community Medicine, University of Bristol, Canynge Hall, 39 Whatley Road, Bristol, BS8 2PS, UK

**Keywords:** Solidarity, Justice, Recognition, Ethics, Liberalism, Communitarianism

## Abstract

Solidarity has for a long time been referred to as the core value underpinning European health and welfare systems. But there has been debate in recent years about whether solidarity, with its alleged communitarian content, can be reconciled with the emphasis on individual freedom and personal autonomy. One may wonder whether there is still a place for solidarity, and whether the concept of justice should be embraced to analyse the moral issues regarding access to health care. In this article, I will answer this question by analysing the normative foundations of the concept of justice, followed by a deeper examination of the concept of solidarity in continental philosophy. More specifically, I will compare the philosophical traditions rooted in Kant (with emphasis on autonomy and individual rights) to approaches rooted in Hegel (with emphasis on individual relations of recognition). In addition, I will present the work of Avishai Margalit on the decent society to criticize a predominantly liberal approach to access to health care. The importance of solidarity lies particularly in its emphasis on relational aspects and the role of recognition in care practices, which are usually ignored in liberal approaches to justice. However, the article will argue that solidarity is not an alternative to a rights-based concept of justice, but must be considered as a necessary complement to it.

The concept of solidarity has for a long time been neglected in the field of bioethics. The importance of relationships and responsibility in health and social care, as suggested by the term ‘solidarity’, perhaps does not fit well with the liberal views that have dominated bioethics since its inception in the late 1960s. However, growing criticism of liberalist models in bioethics, along with increased emphasis on social and relational approaches—as for example in feminist bioethics—have drawn the concept of solidarity into the centre of theoretical debate. A special issue of the journal *Bioethics* [1] on the contribution of solidarity to bioethics, as well as the report *Solidarity* published by the Nuffield Council on Bioethics [2], may be seen as signs of this emerging interest. This special issue of the journal *Theoretical Medicine and Bioethics* on the relation between solidarity and autonomy is another sign of this interest.

While solidarity is put forward as an alternative to autonomy, or an individualised concept of autonomy, the theoretical status of the concept remains unclear. In sociology, solidarity has acquired a clear definition, namely, the degree of social cohesion in a group or society whereby individuals, because of various motivations, are willing to serve and promote the collective interest of the group or of society. However, as a bioethical concept, solidarity must have a theoretical status independent of its sociological interpretation: the fact that individuals are willing to support the greater good does not mean that they *should* be doing so as a moral imperative. A moral understanding cannot be based on sociological theory as that would be a naturalistic fallacy, which is an important problem, for example, in empirical bioethics. If solidarity is to play a role in bioethics, a philosophical understanding of this concept is necessary. In this article, I will try to contribute to this philosophical understanding by analysing the role of solidarity as a normative principle in health care policy and compare it to the concept of justice. Though justice is the ruling concept by which to analyse distributive policies in health care, it fails to capture the relational aspects of health care practices. I will argue that solidarity is needed to promote the relational aspects of health care which are ignored and can even be undermined by policies based on justice only.

## Solidarity in health care

The idea of solidarity is commonly used in European countries which have a tradition of national insurance systems that provide security against the impact of disease, old age, and unemployment. The origin of the concept can be found in local initiatives in the nineteenth and early twentieth centuries to arrange small systems of social security and medical support [3]. These arrangements were gradually taken over by the state, which set up a system of financing and provision of social and medical support. Solidarity was eventually enforced by the state by way of compulsory payments of an insurance premium in exchange for financial support in case of medical need or social distress. The state enforcement of solidarity came about by the public insurance system set up by the German Chancellor von Bismarck in the 1880s to compensate workers for the impact of accidents, disability, and illness. This idea of compulsory health care insurance based on solidarity was later followed by a range of other countries on the continent, like Belgium, France, Austria, Switzerland, and the Netherlands.^1^

In health and social care, solidarity has come to mean that everyone makes a fair financial contribution to a collectively organised and compulsory insurance system that guarantees equal access to health and social care services for all members of society. However, though prudence and self-interest are strong motivations in the acceptance of compulsory solidarity, there is also an understanding of solidarity as a feeling of responsibility and a motivation to support those who are in need of care but not able to pay for it [4]. This element of solidarity was typical for local initiatives of mutual support as they developed in the nineteenth century. For a long time, it was an important reason for individuals to support the health and social care arrangements as they developed in the context of the welfare state. For many Europeans, ‘solidarity’ expressed a feeling of togetherness and commitment to the common good, and it was seen as superior to the liberal emphasis on individualism and free choice in countries like the United States.

However, there is now concern about whether the feeling of mutual responsibility is still as strong as it was in the past. Due to the modern emphasis on individual autonomy and cultural heterogeneity, solidarity with other members of society has become more difficult to justify. Solidarity is increasingly defined as *interest solidarity*, meaning, individuals pay their financial contributions to the health and social care system merely because they have an interest to do so. They see their contributions as an ‘investment’ in the health care system in the expectation that they will be helped in situations of need [5]. These expectations are being increasingly challenged as health and social care systems are confronted with increased pressures from an ageing population and medical care costs. While, in the past, the collective responsibility resulted in a universal safety net for illness and disability provided by the state, there is growing consensus that such an unlimited and generous interpretation of solidarity has become unsustainable. The growing limits on benefits and the increase in individual financial contributions may make it less likely that individuals will indeed receive a ‘safe’ return on their ‘investments’.

## The theory of justice

As soon as solidarity is based on personal interest only, it becomes difficult to differentiate it from the liberal idea of justice in which individual interests are balanced against one another in the context of a social contract. The liberal approach is based on a concept of autonomous individuals negotiating their interests. From this perspective, liberal debates tend to focus on the normative evaluation of the performance of the system in terms of a fair distribution of services. This model is most outspoken in the theory of justice developed by John Rawls [6]. Rawls proposes two principles of justice to evaluate the distribution of social and economic advantages in a society. According to Rawls, these principles would be accepted by individuals who deliberate about a just distribution behind a ‘veil of ignorance’, that is, without knowing their own particular circumstances or social position. The first principle of justice is that ‘each person is to have an equal right to the most extensive basic liberty compatible with a similar liberty for others’. The Second Principle is that ‘social and economic inequalities are to be arranged so that they are both (a) reasonably expected to be to one’s advantage, and (b) attached to positions and offices open to all’ [6].

Rawls argues that each member of society has inviolable rights that the greater good of society cannot override: ‘Justice denies that the loss of freedom for some is made right by a greater good shared by others…. [I]n a just society the basic liberties are taken for granted and the rights secured by justice are not subject to political bargaining or to the calculus of social interests’ [6, p. 28]. For Rawls, justice is fairness, meaning, each individual has basic liberties and rights which cannot be overruled by other social interests or by the utilitarian principle of ‘the greatest good for the greatest possible number’. The principles as agreed in the original position are based on a fair procedure: they require equal liberties for all and permit only those economic and social inequalities which are to each person’s interests [6, p. 33].

The principles of justice put limits on individual satisfactions and conceptions of the good as they specify the boundaries of what individuals and society need to respect. This perspective then leads to Rawls’s famous words that in justice as fairness, ‘the concept of the right is prior to that of the good’ [6, p. 31]. A just social system provides the framework of rights and opportunities in which individuals develop their aims and satisfactions. This principle, the priority of the right over the good, is a central feature in Rawls’s conception of justice, but it is also the principle which has been heavily criticized, particularly by communitarian authors [7].

An important element of Rawls’s theory of justice is the development of an index of primary goods which can serve as a yardstick for assessing the extent to which inequalities can be justified or not. According to Rawls, primary goods are things a rational man would prefer to have for himself to reach certain goals [6, p. 92]. Rawls’s assumption is that, while all individuals might have different final ends and plans, they all require for their execution certain primary goods, which can be natural or social. Intelligence, wealth, and opportunity are examples of primary goods that will enable individuals to reach ends they would not reach otherwise. Individuals in the original position would want more of those goods as it is in their interest to have them. The precise content of the index of primary goods is defined by what Rawls calls the *thin theory of the good* [6]. It is ‘thin’ as it includes only minimal assumptions which are shared by individuals about what kinds of good are useful to all individual conceptions of the good. The thin theory of the good is distinct from a *full theory of the good* insofar as it provides only for those things that can be agreed upon as minimally required, while the full theory includes particular conceptions of the good based on the values and ends of individuals. According to Rawls, it is difficult to develop a full theory of the good because of the differences between individual values and the problem of reconciling them by way of consensus. However, we are able to develop a thin theory of the good on the basis of rational preferences for primary goods [6].

## Justice and benevolence

Though the deliberations behind the veil of ignorance are led by rational and impartial motivations, these deliberations take place on the basis of some sense of benevolence towards the less well-off in society. Michael Sandel argues that the obligations and principles to which the individuals are supposed to agree are, in the end, rooted in certain conceptions of our obligations to others [8]. However, there is the risk that justice can become so dominant and restricting that it destroys the relations of benevolence in which it is rooted. Sandel gives the example of the (ideal) family in which relations are governed by spontaneous affection and circumstances of justice prevail only to a small degree [8]. Individual rights are seldom invoked due to a spirit of generosity within the family. However, when the family is wrought with dissension and individual interests grow divergent, the circumstances of justice become more acute. Affection and spontaneity give way to demands for fairness and observance of rights. A worrying situation arises when, from a misplaced sense of justice, individuals take on a calculative attitude and claim a precise share of the expenditures or income. According to Sandel, in such a situation, individuals may lose the spontaneous mutual benevolence which may be typical for the previous situation. There may be no injustice, but the exercise of justice in such an inappropriate way may have brought about an overall decline in the moral character of the association: ‘justice in this case will have been not a virtue but a vice’ [8, p. 35].

Rawls’s ideas certainly cannot be characterized in terms of ‘vice’ and minimal benevolence toward those in unfortunate circumstances. His idea of social order is an egalitarian scheme that is supposed to be to the benefit of all [6]. However, there is a tendency in liberalism to restrict standards of provisions and obligations for support to a minimum. For example, opportunity based egalitarianism, as developed by Ronald Dworkin, argues that redistribution of wealth is not a matter of justice but of choice and personal responsibility [9]. This theory has been embraced by advocates of the ‘New Right’ [10], who argue that if there are job opportunities available, individuals who refuse to work should receive only limited benefits. Such people do not lack the opportunities the employed have, and there is no reason or argument from justice to transfer benefits from the employed to the voluntary unemployed. Opportunity egalitarianism, and particularly its interpretation by the ‘New Right’ [10], has been accused of an attitude of ‘coldness’ in which social support is based on a scrutiny of people’s needs and the opportunities that have been open to them [11, 12]. In the New Right, elements of commonality and reciprocity are narrowed down to rational calculations based on a negative identification with the ‘receiver’ of goods. As soon as others are perceived as making extravagant claims or are otherwise deemed ‘undeserving’, there are fewer reasons for distributing a high standard of goods. People need very few examples of others making ill use of the system to reduce their support. One can also think of decreasing support for individuals who do not behave responsibly because of unhealthy life styles. From the perspective of opportunity egalitarianism, such individuals are abusing access to health services and do not ‘deserve’ to get their health care costs paid by the public health care system. Justice in this context means that that the contributions by the ‘givers’ must be matched by the ‘right’ behavior of the ‘recipients’ of health care.

## Humiliation

Opportunity egalitarians argue that if there is an opportunity to work, there is no reason why the employed should transfer resources to the voluntary unemployed or the ‘undeserving poor’. According to Jonathan Wolff this suspicion results in authorities or institutions collecting various data from individuals as a condition for welfare or benefit payments. The purpose of such practices is to verify the extent to which the fortune of individuals is the result of their choices and how much can be attributed to their circumstances. Wolff argues that this argument results in disrespectful and humiliating practices, ‘subjecting the poor to a level of scrutiny and control not experienced by the better off’ [13, pp. 121–122]. Wolff calls this a process of ‘shameful revelation’ in which they are treated with rudeness and humiliation [13, p. 109].

Wolff discusses scrutiny and ‘coldness’ in the context of unemployment and welfare practices, but this critique can be extended to health and social care. Access to long-term care support services, for example, is becoming more difficult as a result of financial pressures on the system, and is increasingly based on strict assessments of the needs of individuals and the possible contributions by families. Home care services are being reduced to short-term visits of fifteen minutes per visit, while families are required to prove that they are not able to provide the support for their family members at home. In the Netherlands, for example, applications for home care support are scrutinized by city councils or central agencies in order to determine whether families (particularly spouses) have the capacity to take care of their loved ones themselves and whether they are not abusing the system. Care and home help are separated, with families made more responsible for household tasks. Access to long-term care facilities in institutions like nursing homes is likewise severely restricted, as admissions are also based on a scrutiny of the potential within families to deliver care at home.

In his book, *The Decent Society*, Avishai Margalit makes a distinction between a just society and a decent society [14]. According to Margalit, a decent society is one in which institutions are designed to prevent the humiliation of people by other people. Humiliation is defined by Margalit as ‘any behavior or condition that constitutes a sound reason for a person to consider his or her self-respect injured’ [14, p. 9]. Institutions have an inherent tendency to humiliate people, for example, by rejection, exclusion, paternalism, and denial of rights. Margalit notes that many institutions of the welfare state force their beneficiaries to go through humiliating procedures in order to obtain their rightful provisions. In contrast, a decent society is one that cares that the institutions themselves do not operate in a humiliating way. It is a society that fights conditions that constitute a justification for its dependents to consider themselves humiliated [14].

According to Margalit a *just* society is not necessarily a *decent* society. There is no doubt that the spirit of a just society, based on Rawls’s principles of liberty and justified difference, conflicts essentially with a non-decent society [14]. But, as Margalit argues, a Rawlsian just society and distribution of primary goods can still contain humiliating institutions, particularly in terms of the *procedures* by which goods are distributed to needy individuals. The distribution of services may be efficient and just, but can still reflect a lack of compassion and an expression of vindictiveness.

According to Rawls, part of the primary goods is the sense of value that people have of themselves—the sense that their life plans are worthy of realization—as well as the confidence to carry out their plans. Self-respect is the most basic primary good, as without it, there is no point in doing anything whatsoever. Rawls argues that the parties in the original position would wish to avoid at almost any cost the social conditions that undermine self-respect. Rational people wanting to establish a just society will do everything to avoid creating humiliating institutions or conditions, since these would diminish the most basic primary social good. One can accept differences in the distributions of some of the primary goods, but there is no room for any inequality in the distribution of self-respect [14]. If humiliating means damaging people’s self-respect, it should be clear that a society that does not humiliate its members is a necessary condition for a just society.

## Communitarian critique

The individualistic tendencies of liberalism have been strongly criticized by so-called communitarian authors who argue for a stronger awareness of the importance of the community in the life of individuals. The most important element of the communitarian critique is that liberalism presents the individual as an autonomous and independent entity before it engages with others in social practices: individuals are not seen as defined by their membership in communities or practices, but as free to decide to be, or not, a part of any such community, whether religious, political, sexual, or else. So, the problem of liberalism, according to communitarians, is that it presents individuals as disconnected from social practices and communities and refuses to see that their identity as individuals, their well-being, and their values are constituted by and in their participation in these communities [15]. Self-determination and individual freedom can only be exerted within social practices and with reference to the common good. The language of rights of liberal philosophy separates individuals in an artificial way from the social practices in which they are embedded.

Communitarian authors generally criticize contemporary individualism, and particularly the individualism of the market, in which individuals seem to be interested only in chasing their own claims and interests and not in feeling any responsibility for the needs of others [15]. In the view of communitarian thinkers, individualization is a morally doubtful process that tends to undermine the organic ties in society and, therewith, the social responsibilities of individuals. Some communitarians refer to traditional societies in which individuals found moral orientation in the values of family, school, church, and cultural and political associations. They display a nostalgia for the communal ties of past societies characterized by the word ‘*Gemeinschaft’* a term introduced by the sociologist Ferdinand Tönnies (1855–1936) to describe the strong communal ties of societies in the Middle Ages and other pre-modern societies [16]. The strong social cohesion of the *Gemeinschaft* is opposed to the emphasis on the individual and the lack of communal ties in the modern *Gesellschaft*. While the *Gemeinschaft* can be typified by strong mutual bonds and feelings of togetherness, the individuals in the *Gesellschaft* see society merely as instrumental to their own personal ends. Apart from the nostalgic and moralistic atmosphere that pervades much of its discourse, however, it is not clear how communitarianism resolves the problem of how to reach consensus on a societal level concerning what kind of life we should live. Liberal critics fear that the search for commonality as the basis for public policy will result in infringements upon the liberties of individuals and to a ‘totalitarian impulse’.

## Recognition of difference

The idea of solidarity, according to which individuals give up their own interests to serve the common good, seems to fit very well with communitarianism and its emphasis on the importance of society and the social group. However, communitarianism is very much based on a restrictive interpretation of the group in terms of social cohesiveness and exclusiveness: ‘us’ against ‘them’. In such a notion, it is difficult to acknowledge the importance of individuality and autonomy. Moreover, the range of individual differences and of their expression in different identities is restricted by the effort to maintain the unity of the group.

In contrast, modern theories of solidarity try to reconcile the recognition of individual differences with an inclusive interpretation of solidarity. Jody Dean argues that we need to create a communicative ‘we’ by referring to a ‘third’ as a way to create an inclusive community [17]. She refers to the concept of the ‘generalized other’ introduced by the sociologist George Herbert Mead (1863–1931) as a way to organize and formulate the expectations of a group with respect to certain social roles and identities in a group. Dean opens the way for an interpretation of solidarity as a communicative practice in which the individuals create a ‘we’ by reflecting on expectations regarding the generalized other. In this process, identities are affirmed and recognized as different ways to meet those expectations. Dean calls this *reflective solidarity* to describe the difference with conventional solidarity.

Axel Honneth comes to a comparable understanding of solidarity and the recognition of difference. Where modern law provides a medium for the recognition of universal rights, solidarity requires a medium to express the characteristic differences between individuals. Like Dean, Honneth refers to the work of Mead to argue for a ‘framework of orientation’ at the societal level in which ‘those ethical values and goals are articulated that, taken together, comprise the cultural self-understanding of a society’ [18, p. 122]. Such a framework can serve as a ‘system of reference’ for the appraisal of individual personality features. According to Honneth, the cultural self-understanding of a society provides the criteria for the social esteem of persons because their abilities and achievements are judged in an intersubjective process according to the degree to which they help to realize culturally defined values.

Solidarity in Honneth’s work then means an interactive relationship in which individuals mutually sympathize with each other’s different ways of life because they esteem each other in reference to a shared value horizon. While the group is the first instance for such recognition of individual differences, and for the self-esteem resulting from it, such solidarity can be extended to other members of society [18]. One can speak of *societal* solidarity to the extent that every member in society is in a position to esteem himself or herself in relation to a shared value horizon. Honneth and Dean distance themselves from the traditional concepts of solidarity as put forward by communitarian authors, in which the values of the group leave little room for personal development and individual differences in relation to life style, sexual orientation, religion, or race. In a modern version of solidarity, individual differences are recognized, but not just because of the differences only: the value horizon (Honneth) or ‘hypothetical third’ (Dean) provides a social context for the development of self-esteem. This idea goes beyond the liberal idea of the unencumbered individual who is free to make any choices he or she sees as important, and for whom society is a hindrance, not a condition for personal development and self-esteem. This solidarity in the sense of mutual recognition is not the solidarity of the ‘us’ against ‘them’: it is a sense of brotherhood but one that connects a concern for the well-being of the other with the universality of human rights and the protection of dignity. It is not an *exclusive* solidarity of the group or class, but an *inclusive* solidarity that promotes self-esteem by way of solidarity, self-respect, and protection of rights [18].

## Solidarity as a relational concept

As discussed at the beginning of this article, solidarity is nowadays mainly viewed from the perspective of self-interest: individuals are prepared to serve the collective interest because they expect a return when they are in need of support and medical assistance. However, according to some authors, solidarity should be more than just a ‘shallow coalition’ based on common interests [19]. For example, Rahel Jaeggi argues that solidarity expresses a deeper commitment than is necessary for such a coalition. Many attitudes of solidarity do not seem to be directed to simple self-interest or strategic calculations. Solidarity also refers to relations of support and understanding between individuals engaged in cooperative practices. Acting out of solidarity means ‘standing up for each other because one recognises one’s own fate in the fate of the other’ [19, p. 291]. As a moral concept solidarity implies a sense of non-calculating cooperation based on identification with a common cause. This interpretation of solidarity as non-instrumental cooperation connects this concept to Hegel’s idea of *Sittlichkeit* or ‘ethical life’. Jaeggi refers to Michael Theunissen, who defines ethical life as ‘those conditions in which the individual first and foremost finds his own self’ ([20] quoted in [19, p. 295]). According to Jaeggi, individuals realise themselves by connecting to those kinds of relations that are intersubjective conditions of self-realisation: the ‘Other’ is not the limitation but the pre-condition of my freedom.

Hegel introduced the concept of *Sittlichkeit* as distinct from Kant’s concept of *Moralität*: while *Sittlichkeit* refers to the relations of recognition, *Moralität* refers to abstract rights and duties [18, 21]. In the Kantian tradition, justice is interpreted as a matter of universal duties between individuals that can be justified on the basis of rational deliberations. Honneth argues that Kant’s concept of the dignity of persons respects and protects human rights, but that solidarity promotes self-esteem and recognition of oneself [18]. Solidarity is one of the three ‘patterns of recognition’ which are essential to self-realization: love, rights, and solidarity [18]. *Love* is the relationship in which individuals build up trust and self-confidence, starting with the relationship between mother and child. *Rights* express the recognition of and respect for persons as agents capable of acting on the basis of reasons, as morally responsible persons. *Solidarity* is the experience of recognition of one-self as a person with a particular identity in the intersubjective context of mutual recognition. Inspired by Hegel’s idea of *Sittlichkeit*, or ethical life, Honneth’s approach is based on a relational and contextual view of individual development. Solidarity is an essential part of the ethical life as it is a necessary precondition for individual self-esteem. The forms of recognition associated with love, rights, and solidarity provide the intersubjective protection for the process of articulating and recognizing individual identity based on a positive relation to one-self. According to Honneth, both *Moralität* and *Sittlichkeit* are needed: while Kant’s concept of the dignity of persons protects human rights, solidarity promotes self-esteem under the condition of mutual relatedness and the fundamental interdependence of individuals.

## Humanitarian solidarity

Liberal theorists do not regard values of personal commitment and recognition as unimportant, but they do not regard it as the task of society to promote such values or to interfere in personal plans or life forms. The task of society is to enable individuals to facilitate their personal plans on the basis of a principle of fair procedures. The central ethic of procedural liberalism is that of the *right* rather than that of the *good*. There are of course areas in society where individuals bond and share a conception of the good life, like families, friendship, social clubs, and neighborhoods, but on an institutional level, such ties and bonds are irrelevant: institutions like health care or education are collective instruments to help individuals to reach their individual goals and fulfil their life plans.

However, one can argue whether such a limited role for communal ethics is feasible or desirable. According to Charles Taylor, a society is more than just instrumental for individual life plans. It is also a place for common action and common identification with values. Identification with a common cause, or a shared vision of the common good, helps to promote self-discipline and asks the members of a community or nation to do things that they normally would try to avoid [7, p. 193]. A neutral state undermines the shared sense of the common good which is required for citizens to accept the sacrifices of the welfare state [11]. When citizens distance themselves from a shared communal life, they are less inclined to support the welfare arrangements that are based on these common views. In fact, this can lead to a legitimation crisis in which citizens are asked to make increasing sacrifices in the name of justice, while they have less in common with those for whom they are making sacrifices. This is actually the problem in many solidarity based institutions in various European countries where individuals are asked to make high financial and personal sacrifices to deal with the increased pressure on institutions for health and social care due to the ageing of the population and the increased costs of medical care. This support from individuals may dwindle when they are confronted with the prospect of diminishing returns when they eventually need help themselves.

Margalit makes a distinction between *ethics* and *morality*: while ethics is concerned about *thick* relations between individuals, that is, relations that call for actions, morality regulates our *thin* relations which express our concerns for humanity [22, 23].^2^ A society can be ethical but immoral (like Nazi Germany, where there were strong tribal ties but also a lack of humanity), or it can be moral, where people are very concerned about rights but are not interested in relations of compassion. The former leads to a society that is *immoral*, the latter to a society that is *indifferent* [23]. A society that is dominated by liberal principles and rights only risks becoming an indifferent society which is hardly interested in the well-being of fellow citizens. Such a society is particularly at risk of becoming indifferent to its responsibility for vulnerable individuals who cannot help themselves, like people with dementia, learning disabilities, psychiatric problems, and others struggling with a loss of autonomy, failing health, and a lack of security. Care for these vulnerable individuals will not only support their health and social needs, but will also keep them included in our society. Care for them can be regarded as an expression of what Margalit calls *shared humanity* [23]. Failing to provide decent care for vulnerable people does not only express a lack of ethical concern, but is at the same time a violation of the rules of humanity in which respect of human dignity is central.

Solidarity with the vulnerable groups in society can be called *humanitarian* solidarity: this type of solidarity is not based on personal interests but on identification with the values of humanity and responsibility for the other [24, 25]. Humanitarian solidarity combines the principles of *Moralität* by respecting rights and preventing humiliation, and the concerns of *Sittlichkeit* by creating personal support and recognition of individual difference. Humanitarian solidarity is a common cause (Taylor) that goes beyond the self-interest and indifference typical for a society based on liberal rights only. Humanitarian solidarity is a commitment that can define a particular society and should never be abandoned in favor of the rational self-interest of the liberal discourse.

## Conclusion

The concept of solidarity tries to capture the commitment to the well-being of the other by emphasizing the importance of recognition of identities and the promotion of dignity in the context of personal relationships. This is not to say that justice should be discarded in the arrangement of health care policies and practices in favor of solidarity; solidarity does not attempt to offer an alternative for distributive justice. But solidarity must be regarded as an important corrective to arrangements of health care practices that are based on a just distribution of goods only. Health care policies and arrangements should go beyond merely meeting needs and rights by exploring how people’s personal dignity and sense of belonging can be sustained within relations of recognition, reciprocity, and support.

## Footnotes

1 The United Kingdom and the Scandinavian countries followed a different path where health care and social support came to be provided on the basis of a taxation scheme (the so-called *Beveridge* system, named after the British economist and social reformer William Beveridge). Though solidarity is not explicitly referred to in these tax-based health care systems, many of their policies have similar social goals as the insurance systems based on the principle of solidarity.

2 This distinction by Margalit reflects in many ways the distinction between *Moralität* and *Sittlichkeit* mentioned above.
